# A phase I study of single-agent BEZ235 special delivery system sachet in Japanese patients with advanced solid tumors

**DOI:** 10.1007/s00280-018-3725-2

**Published:** 2018-11-16

**Authors:** Masanori Toyoda, Koichiro Watanabe, Taro Amagasaki, Kazuto Natsume, Hiromi Takeuchi, Cornelia Quadt, Kuniaki Shirao, Hironobu Minami

**Affiliations:** 1 Division of Medical Oncology/Hematology, Department of Medicine, Kobe University Hospital, Kobe University Graduate School of Medicine, 7-5-1 Kusunoki-cho, Chuo-ku, Kobe, 650-0017 Japan; 2 Department of Medical Oncology and Hematology, Oita University Faculty of Medicine, Oita, Japan; 3 Novartis Pharma K.K, Tokyo, Japan; 4 Novartis Pharma AG, Basel, Switzerland; 5 Present Address: Department of Medical Oncology, Kouseiren Tsurumi Hospital, Oita, Japan

**Keywords:** PI3K, mTOR, Phase I, BEZ235, Inhibitor

## Abstract

**Purpose:**

BEZ235 is a dual kinase inhibitor of phosphatidylinositol 3-kinase (PI3K) and mammalian target of rapamycin, which are key components of the PI3K pathway. This was an open-label, multicenter, dose-escalation, phase I study of single-agent BEZ235 in Japanese oncology patients to determine the maximum tolerated dose (MTD) of BEZ235 based on dose-limiting toxicities (DLTs).

**Methods:**

Dose escalation was guided by a standard 3 + 3 method and was based on DLTs observed in Cycle 1 and other safety, pharmacokinetic, and pharmacodynamic information. A total of 35 adult Japanese patients with advanced solid tumors received BEZ235 according to once daily (qd; *n* = 27) or twice daily (bid; *n* = 8) dosing schedules.

**Results:**

Two DLTs, namely, allergic reaction and thrombocytopenia, were observed at 1200 and 1400 mg qd, respectively, while liver dysfunction was reported as a DLT at 400 mg bid. The most common adverse events suspected to be related to BEZ235 in both dosing schedules were diarrhea, nausea, decreased appetite, stomatitis, and thrombocytopenia.

**Conclusions:**

Although the MTD was not established, the maximum clinically tolerable dose was determined to be 1200 mg because two out of six patients required dose reduction in Cycle 2. The recommended dose was determined to be 1000 mg qd, which was comparable with the results of the first-in-human BEZ235 study in Western patients with advanced solid tumors (NCT00620594). Additionally, the tolerability of BEZ235 400 mg bid in Japanese oncology patients was confirmed in this study.

**ClinicalTrials.gov identifier:**

NCT01195376.

**Electronic supplementary material:**

The online version of this article (10.1007/s00280-018-3725-2) contains supplementary material, which is available to authorized users.

## Introduction

The phosphatidylinositol 3-kinase (PI3K) /mammalian target of rapamycin (mTOR) pathway is a key signal transduction systems linking multiple receptor tyrosine kinases and oncogenic proteins to essential cellular functions such as cell growth, survival, motility, and metabolism [1]. Important components of the signaling cascade include AKT, mTOR [2], and phosphatase and tensin homolog (PTEN), a negative regulator of PI3K [3]. Several lines of evidence suggest that constitutive PI3K activation is a critical step in mediating the transforming potential and growth stimulating activity of various oncogenes and tumor suppressors, thereby contributing to the onset and growth of many solid tumors as well as tumors of the hematopoietic system [4, 5].

At low exposures, BEZ235 is a catalytic inhibitor of mTOR, inhibiting both mTORC1 and mTORC2 kinases, whereas at high exposures, which were expected at the doses tested in this study, BEZ235 inhibits mTOR as well as all class I PI3Ks. The antitumor activity of BEZ235 was demonstrated in preclinical studies [6].

Several formulations of BEZ235, such as a hard gelatin capsule formulation and a special delivery system (SDS) formulation provided as capsules or granules in a sachet, were investigated in the first-in-human (FIH) study of BEZ235 [7]. The SDS formulations were developed to decrease pharmacokinetic (PK) variability and improve bioavailability. However, at the doses of 400 mg and higher tested in this study, the SDS capsule still showed high inter-patient variability, but the PK profile of the SDS sachet formulation appeared more consistent. Here, we report the results of a phase I study to investigate the safety, tolerability, preliminary efficacy, PK, and maximum tolerated dose (MTD) of BEZ235 SDS granules in a sachet formulation in Japanese patients. To reduce the risk of gastrointestinal toxicities observed with the once daily (qd) dosing regimen, twice daily (bid) dosing of BEZ235 was also investigated.

## Materials and methods

### Study oversight

This study was designed as a phase I, multicenter, open-label trial in which BEZ235 SDS sachet was administered orally as a single agent on a continuous qd or bid dosing schedule in Japanese patients with advanced solid malignancies (ClinicalTrials.gov Identifier: NCT01195376). The study was designed by the sponsor (Novartis Pharmaceuticals Corporation) and performed in accordance with the principles of Good Clinical Practice. The protocol was approved by an Institutional Review Board at each institution and the study was conducted according to the ethical principles of the Declaration of Helsinki. Informed consent was obtained from all individual participants included in the study.

### Patient selection

Key inclusion criteria were as follows: patients with histologically confirmed, advanced, unresectable solid tumors who have progressed on (or were not able to tolerate) standard therapy or for whom no standard anticancer therapy existed; at least one measurable lesion as defined by the Response Evaluation Criteria in Solid Tumors (RECIST) v1.0; age ≥ 20 years; Eastern Cooperative Oncology Group performance status (ECOG PS) 0–2; and life expectancy of at least 12 weeks.

Key exclusion criteria were as follows: patients with brain tumor and/or brain metastases, with signs/symptoms potentially attributable to brain metastases or if brain lesions could not be ruled out by radiologic imaging, fulminant disease including fulminant hepatitis, prior treatment with a PI3K inhibitor, acute or chronic liver disease, clinically relevant renal disease, acute or chronic pancreatitis, history or presence of interstitial lung disease (ILD), peripheral neuropathy ≥ Common Terminology Criteria for Adverse Events (CTCAE) grade 2, and unresolved diarrhea ≥ CTCAE grade 2.

### Study objectives

The primary objective was to determine the MTD of BEZ235 as a single agent based on the incidence rate of dose-limiting toxicities (DLTs) when administered orally. The secondary objectives were to assess the safety and tolerability of BEZ235, characterize its PK profiles, evaluate preliminary efficacy, and assess potential treatment effects on the pharmacodynamic (PD) biomarkers of the PI3K/mTOR pathway.

### Study design and treatment plan

BEZ235 was administered orally as a single agent on a continuous qd or bid dosing schedule. The starting dose level for the first cycle (28 days) was set as 400 mg/day qd, based on the FIH BEZ235 study in Western patients (NCT00620594). In this study, no study drug-related ≥ grade 2 adverse events (AEs) were observed during Cycle 1 with the SDS capsule at 400 mg qd. At the 800 mg qd dose level, one DLT (grade 3 fatigue) was observed and at the 1000 mg qd dose level, two DLTs (grade 3 fatigue and skin rash, which required more than 7 days treatment interruption) were observed. The MTD for the BEZ235 SDS capsule was established at 1000 mg qd [8]. Furthermore, in the previous study, a granule formulation (BEZ235 SDS sachet) was developed because of difficulties in swallowing the large size capsules of the investigational drug. The ingredients of this new formulation, which are dispensed as granules contained within a sachet, are essentially the same as the capsule. Therefore, it was anticipated that the PK profiles would not differ to an extent that would significantly affect the dosing of the sachet formulation. The starting dose of 400 mg qd was half of the dose level of the BEZ235 SDS capsule with the first DLT and also less than half of the MTD of the BEZ235 SDS capsule. The standard 3 + 3 method for dose escalation was used to establish the MTD of BEZ235. Decisions regarding dose escalation were based on the safety profile, particularly DLTs, observed in the first cycle of treatment for each patient.

### Safety assessments

Safety assessments consisted of monitoring and recording of all AEs, including laboratory abnormalities and vital signs. DLTs were defined as an AE or a laboratory abnormality assessed as related to the study drug and occurring within the first 28 days of treatment (Cycle 1), as defined in Supplemental Table S1.

### Response assessments

Tumor lesions were assessed by the investigators according to the RECIST guidelines, v1.0. Patients underwent screening using computed tomography (CT) or magnetic resonance imaging (MRI) scans. Screening was performed within 28 days of the first dose. The post-baseline RECIST assessments were performed every two cycles and then at the end of treatment.

### Pharmacokinetics

Blood samples for plasma concentration–time profiles of BEZ235 and its relevant metabolites were collected from all patients. The plasma samples were assayed for BEZ235 concentrations using a validated liquid chromatography–tandem mass spectrometry assay (LC–MS/MS). The PK parameters were determined by non-compartmental analysis using Phoenix WinNonlin 6.2^®^ (Pharsight, Mountain View, CA). The following PK parameters were summarized as the primary PK parameters for BEZ235 and its metabolite(s): AUC_last_, AUC_0 − 24_, AUC_0 − 12_, AUC_inf_, *C*_max_, and *T*_max_.

### Biomarkers

Normal skin samples were collected at baseline and the end of Cycle 1 to assess the effect of BEZ235 on the PI3K/mTOR pathway based on the level of S6 phosphorylation (pS6). Tumor samples were analyzed at a qualified laboratory for confirming the gene status of PIK3CA and PTEN.

## Results

### Patient characteristics

Between 06-Oct-2010 and 03-Jul-2013, 35 patients were enrolled and received at least one dose of BEZ235 with either the qd (*n* = 27) or bid (*n* = 8) dosing schedule. The baseline demographic and patient characteristics data are presented in Table 1. Patients with various types of cancers were enrolled, and all patients except one patient with carcinoid of the rectum were heavily pretreated before enrollment.

**Table 1:** Patient demographics and disease characteristics based on treatment group

Characteristics	BEZ235 qd (*N* = 27)						BEZ235 bid
	400 mg (*n* = 3)	800 mg (*n* = 3)	1000 mg (*n* = 8)	1200 mg (*n* = 6)	1400 mg (*n* = 7)	All patients (*N* = 27)	400 mg (*N* = 8)
Age, years, median (range)	65 (52–69)	62 (61–74)	59 (31–70)	65 (37–67)	55 (38–74)	62 (31–74)	50 (36–74)
Sex, *N* (%)							
Male	0	2 (66.7)	8 (100)	4 (66.7)	2 (28.6)	16 (59.3)	4 (50.0)
Female	3 (100)	1 (33.3)	0	2 (33.3)	5 (71.4)	11 (40.7)	4 (50.0)
ECOG PS, *N* (%)							
0	2 (66.7)	0	4 (50.0)	3 (50.0)	2 (28.6)	11 (40.7)	3 (37.5)
1	1 (33.3)	3 (100)	4 (50.0)	3 (50.0)	5 (71.4)	16 (59.3)	5 (62.5)
Primary site of cancer, *N* (%)							
Rectum	0	0	2 (25.0)	1 (16.7)	2 (28.6)	5 (18.5)	1 (12.5)
Lung	0	0	1 (12.5)	0	2 (28.6)	3 (11.1)	0
Pancreas	0	1 (33.3)	0	0	1 (14.3)	2 (7.4)	2 (25.0)
Colon	0	0	0	2 (33.3)	0	2 (7.4)	1 (12.5)
Bone	1 (33.3)	0	0	0	1 (14.3)	2 (7.4)	1 (12.5)
Soft tissue	0	0	2 (25.0)	0	0	2 (7.4)	0
Stomach	0	0	1 (12.5)	0	0	1 (3.7)	2 (25.0)
Other	1 (33.3)	2 (66.7)	1 (12.5)	1 (16.7)	1 (14.3)	6 (22.2)	1 (12.5)
Unknown	1 (33.3)	0	1 (12.5)	2 (33.3)	0	4 (14.8)	0
Number of prior regimens, *N* (%)							
0	0	0	0	0	1 (14.3)	1 (3.7)	0
1–2	3 (100)	2 (66.7)	2 (25.0)	4 (66.7)	3 (42.9)	14 (51.9)	3 (37.5)
3–4	0	0	6 (75.0)	1 (16.7)	1 (14.3)	8 (29.6)	3 (37.5)
≥ 5	0	1 (33.3)	0	1 (16.7)	2 (28.6)	4 (14.8)	2 (25.0)

*bid* twice daily, *ECOG PS* Eastern Cooperative Oncology Group Performance Status, *qd* once daily

### Safety

Among the 27 patients enrolled in the qd group, 24 patients were included in the dose-determining analysis set (400 mg, *n* = 3; 800 mg, *n* = 3; 1000 mg, *n* = 6; 1200 mg, *n* = 6; and 1400 mg, *n* = 6). Three patients were excluded because of the following reasons: did not receive the required minimum number of BEZ235 doses in Cycle 1 due to disease progression (1000 mg group), AE that did not meet the DLT criteria (1000 mg group), and withdrawal of consent (1400 mg group). One out of the six evaluable patients in the 1200 and 1400 mg qd groups each experienced DLTs of grade 2 allergic reaction and grade 4 thrombocytopenia, respectively (Table 2). In one patient who experienced allergic reaction in the 1200 mg qd group, the reaction was accompanied with grade 1 fever and grade 3 rash that spread suddenly when the patient restarted the study treatment after interruption. The study treatment had to be discontinued in this patient due to this event. Therefore, this allergic reaction was considered as a DLT, although it was not prespecified in the protocol definitions of DLT (Online supplement Table S1).

**Table 2:** Summary of patient treatment and DLTs

Regimen	Dose (mg)	Full analysis set	Dose-determining analysis set	Number of patients who experienced a DLT	DLT
BEZ235 qd (*N* = 27)	400	3	3	0	–
	800	3	3	0	–
	1000	8	6	0	–
	1200	6	6	1	Grade 2 allergic reaction
	1400	7	6	1	Grade 4 thrombocytopenia
BEZ235 bid (*N* = 8)	400	8	6	1	Grade 2 liver dysfunction

*bid* twice daily, *DLT* dose-limiting toxicity, *qd* once daily

Even though a DLT occurred in only one out of the six patients at 1400 mg qd, the dose was not escalated to more than 1400 mg, considering that grade 3/4 thrombocytopenia was observed in one patient at 1400 mg. Furthermore, dose interruption due to AEs was required in six patients, and three patients required dose reduction in early cycles. Therefore, the MTD, which is the highest drug dose that causes DLT in no more than one out of six patients in Cycle 1, was not reached. While the rates of DLTs were similar at both 1200 mg qd and 1400 mg qd, the safety of 1400 mg qd was considered less favorable. As described above, severe (grade 3/4) thrombocytopenia, dose interruptions in Cycle 1 and dose reductions after Cycle 1 were more frequently observed in patients treated with 1400 mg than 1200 mg. Therefore, 1200 mg was determined as the maximum clinically tolerable dose of BEZ235 in a qd dosing schedule, while 1000 mg was determined as the recommended dose (RD) because it was the highest dose that did not cause any DLT.

Eight patients were treated with BEZ235 at a dose of 400 mg bid (Table 2). Of these, three patients were excluded from the dose-determining analysis set because they had not received the required minimum number (i.e., bid for 21 days) of BEZ235 doses in Cycle 1 for DLT evaluation due to an AE that did not meet the DLT criteria. However, one out of the three excluded patients was considered as clinically relevant for the evaluation of DLT. This patient suspended drug administration from the second dosing of Cycle 1 Day 14 to the first dosing of Day 21, resulting in a total of 14 skipped doses (corresponding to 7 days). Therefore, this patient was considered as evaluable for the assessment of safety and tolerability based on the discussion between the investigators and the sponsor. A total of six patients, including this patient, were evaluated for the determination of DLTs. Only one out of six patients in the 400 mg bid cohort experienced a DLT of grade 2 liver dysfunction (Table 2). This patient had adenocarcinoma of the rectum with liver metastases and elevated levels of total bilirubin, alkaline phosphatase, and gamma-GTP documented during screening. These values continued to increase when the patient restarted treatment after dose interruption, regardless of dose reduction. Therefore, these abnormal values were considered to be liver dysfunction related to the study drug and were evaluated as a DLT, although each abnormal value did not meet the DLT criteria for hepatic toxicities. In conclusion, 400 mg bid was considered as a tolerable dose.

The median duration of exposure was 56 (2–280) days for the qd and 43.5 (21–115) days for the bid dosing schedules (Table 3). All patients in both dosing groups experienced at least one AE. In the qd group, the most frequently occurring AEs were diarrhea, decreased appetite, nausea, stomatitis, fatigue, and vomiting. In the bid group, the most frequently occurring AEs were decreased appetite, diarrhea, nausea, stomatitis, fatigue, and thrombocytopenia. Most of the patients in both groups experienced diarrhea, decreased appetite, and nausea (Tables 4, 5).

**Table 3:** Overall exposure

Overall exposure	BEZ235 qd						BEZ235 bid
	400 mg (*n* = 3)	800 mg (*n* = 3)	1000 mg *(n* = 8)	1200 mg (*n* = 6)	1400 mg (*n* = 7)	All patients (*N* = 27)	400 mg (*N* = 8)
Exposure (weeks)							
≤ 4	0	0	3 (37.5%)	2 (33.3%)	1 (14.3%)	6 (22.2%)	3 (37.5%)
> 4 to ≤ 8	2 (66.7%)	0	2 (25.0%)	2 (33.3%)	2 (28.6%)	8 (29.6%)	2 (25.0%)
> 8 to ≤ 12	0	0	2 (25.0%)	0	1 (14.3%)	3 (11.1%)	1 (12.5%)
> 12	1 (33.3%)	3 (100.0%)	1 (12.5%)	2 (33.3%)	3 (42.9%)	10 (37.0%)	2 (25.0%)
Duration of study treatment exposure (days)							
*n*	3	3	8	6	7	27	8
Mean	52.7	149.7	48.8	82.3	77.4	75.3	55.9
SD	28.99	31.77	30.78	100.31	52.23	62.36	37.72
Median	44.0	167.0	37.0	39.5	68.0	56.0	43.5
Minimum	29.0	113.0	19.0	16.0	2.0	2.0	21.0
Maximum	85.0	169.0	112.0	280.0	169.0	280.0	115.0

*bid* twice daily, *qd* once daily, *SD* standard deviation

**Table 4:** Frequent AEs (≥ 20% in all patients), regardless of study drug relationship—qd dosing schedule

	BEZ235 qd											
	400 mg (*n* = 3)		800 mg (*n* = 3)		1000 mg (*n* = 8)		1200 mg (*n* = 6)		1400 mg (*n* = 7)		All patients (*N* = 27)	
PT	All grades *n* (%)	Grade ¾ *n* (%)	All grades *n* (%)	Grade ¾ *n* (%)	All grades *n* (%)	Grade ¾ *n* (%)	All grades *n* (%)	Grade ¾ *n* (%)	All grades *n* (%)	Grade ¾ *n* (%)	All grades *n* (%)	Grade ¾ *n* (%)
Total	3 (100.0)	2 (66.7)	3 (100.0)	1 (33.3)	8 (100.0)	4 (50.0)	6 (100.0)	6 (100.0)	7 (100.0)	5 (71.4)	27 (100.0)	18 (66.7)
Diarrhea	2 (66.7)	0	3 (100.0)	0	7 (87.5)	0	6 (100.0)	0	7 (100.0)	1 (14.3)	25 (92.6)	1 (3.7)
Decreased appetite	2 (66.7)	0	3 (100.0)	0	7 (87.5)	0	6 (100.0)	1 (16.7)	6 (85.7)	0	24 (88.9)	1 (3.7)
Nausea	3 (100.0)	0	1 (33.3)	0	7 (87.5)	0	5 (83.3)	0	6 (85.7)	0	22 (81.5)	0
Stomatitis	2 (66.7)	0	2 (66.7)	0	3 (37.5)	0	6 (100.0)	1 (16.7)	5 (71.4)	0	18 (66.7)	1 (3.7)
Vomiting	2 (66.7)	0	1 (33.3)	0	4 (50.0)	0	5 (83.3)	0	5 (71.4)	0	17 (63.0)	0
Fatigue	1 (33.3)	0	2 (66.7)	0	6 (75.0)	1 (12.5)	4 (66.7)	2 (33.3)	4 (57.1)	0	17 (63.0)	3 (11.1)
Rash	1 (33.3)	0	0	0	5 (62.5)	1 (12.5)	1 (16.7)	1 (16.7)	4 (57.1)	0	11 (40.7)	2 (7.4)
Lymphopenia	2 (66.7)	2 (66.7)	1 (33.3)	1 (33.3)	2 (25.0)	2 (25.0)	1 (16.7)	1 (16.7)	4 (57.1)	1 (14.3)	10 (37.0)	7 (25.9)
Neutropenia	0	0	1 (33.3)	0	1 (12.5)	1 (12.5)	2 (33.3)	0	4 (57.1)	3 (42.9)	8 (29.6)	4 (14.8)
Thrombocytopenia	0	0	1 (33.3)	1 (33.3)	4 (50.0)	0	0	0	3 (42.9)	2 (28.6)	8 (29.6)	3 (11.1)
Dysgeusia	0	0	1 (33.3)	0	2 (25.0)	0	3 (50.0)	0	2 (28.6)	0	8 (29.6)	0
Cancer pain	2 (66.7)	1 (33.3)	2 (66.7)	0	2 (25.0)	0	1 (16.7)	0	0	0	7 (25.9)	1 (3.7)
Leukopenia	0	0	1 (33.3)	0	2 (25.0)	1 (12.5)	1 (16.7)	0	2 (28.6)	1 (14.3)	6 (22.2)	2 (7.4)
Pyrexia	1 (33.3)	0	1 (33.3)	0	2 (25.0)	0	2 (33.3)	0	0	0	6 (22.2)	0
Alanine aminotransferase increased	2 (66.7)	0	0	0	0	0	1 (16.7)	0	3 (42.9)	1 (14.3)	6 (22.2)	1 (3.7)
Blood alkaline phosphatase increased	1 (33.3)	0	0	0	2 (25.0)	0	1 (16.7)	1 (16.7)	2 (28.6)	1 (14.3)	6 (22.2)	2 (7.4)

AEs are listed in descending order within PT level in the all patients group

*AE* adverse event, *PT* preferred term, *qd* once daily

**Table 5:** Frequent AEs (≥ 20% in all patients), regardless of study drug relationship—bid dosing schedule

	BEZ235 bid	
	400 mg *N* = 8	
PT	All grades *n* (%)	Grade ¾ *n* (%)
Total	8 (100.0)	7 (87.5)
Diarrhea	7 (87.5)	1 (12.5)
Nausea	7 (87.5)	0
Decreased appetite	7 (87.5)	1 (12.5)
Thrombocytopenia	5 (62.5)	2 (25.0)
Stomatitis	5 (62.5)	0
Fatigue	5 (62.5)	1 (12.5)
Lymphopenia	4 (50.0)	2 (25.0)
Vomiting	4 (50.0)	0
Alanine aminotransferase increased	4 (50.0)	0
Aspartate aminotransferase increased	4 (50.0)	1 (12.5)
Haemoglobin decreased	4 (50.0)	1 (12.5)
Insomnia	4 (50.0)	0
Dry skin	4 (50.0)	0
Rash	4 (50.0)	0
Hypoalbuminaemia	4 (50.0)	1 (12.5)
Cheilitis	3 (37.5)	0
Dysphagia	3 (37.5)	0
Cancer pain	3 (37.5)	2 (25.0)
Blood creatinine increased	3 (37.5)	0
Malaise	2 (25.0)	1 (12.5)
Pyrexia	2 (25.0)	0
Urinary tract infection	2 (25.0)	0
Blood alkaline phosphatase increased	2 (25.0)	0
Gamma-glutamyltransferase increased	2 (25.0)	0
Hyperkalaemia	2 (25.0)	0
Hyponatraemia	2 (25.0)	1 (12.5)
Anxiety	2 (25.0)	0

AEs are listed in descending order within PT level

*AE* adverse event, *bid* twice daily, *PT* preferred term

Grade 3 or 4 AEs were reported in 18 patients (66.7%) in the qd dosing and seven patients (87.5%) in the bid dosing groups. In the qd group, grade 3 or 4 AEs suspected to be study drug-related reported in more than one patient were lymphopenia [four patients (14.8%)], neutropenia and fatigue [three patients (11.1%) each], and thrombocytopenia and rash [two patients (7.4%) each]. In the bid group, grade 3 or 4 AEs suspected to be study drug-related included thrombocytopenia and lymphopenia, which were reported in two patients (25.0%) each and all other grade 3 or 4 AEs occurred in a single patient.

No patient died during the study period, including a 30-day follow-up. Study drug-related serious adverse events (SAEs) were reported for only two patients in the qd dosing group, namely, grade 2 colitis (1000 mg) and grade 2 Pneumocystis jiroveci pneumonia (1400 mg). In the bid dosing group, one patient experienced a study drug-related SAE of grade 4 diarrhea.

The most frequent reason for treatment discontinuation was disease progression [16 patients (59.3%) with qd dosing and seven patients (87.5%) with bid dosing]. In the qd group, seven patients (25.9%) discontinued the study drug due to AEs. In the bid group, an AE leading to study drug discontinuation was reported in one patient (12.5%). This rectal cancer patient who experienced a DLT of grade 2 liver dysfunction discontinued the study drug in consideration of the balance between benefit with the study medicine and the risk of abnormal hepatic function, as the DLT was suspected to be study drug-related.

### Efficacy

All 35 patients were evaluable for response per RECIST v1.0 guidelines. No complete or partial responses (CR or PR) were reported across all dose levels in any of the dosing schedule groups. Stable disease (SD) was observed as the best response in 14 of 27 (51.9%) evaluable patients with a mean duration of study treatment exposure of 75.3 days and 2 of 8 (25.0%) evaluable patients with a mean duration of study treatment exposure of 55.9 days in the qd and bid dosing groups, respectively. One patient with carcinoma of unknown primary who was evaluated as having SD with continued BEZ235 qd treatment for 10 months.

### Pharmacokinetics

In general, the *C*_max_ and area under the curve (AUC) increased with dose on Cycle 1 Day 1 and Day 8, though no clear trend was observed on Day 28. For both the qd and bid regimens, PK exposures on Day 8 were at least threefold higher than on Day 1, whereas exposures on Day 8 and Day 28 were comparable. Based on these findings, the steady-state was expected to be achieved at least on Day 8. At these oncology doses, the inter-individual variability was high, with a coefficient of variation greater than 100% on the AUC and *C*_max_ at most of the visits and doses. Median T_max_ was approximately 4 h across all dose levels and dosing schedules (individual T_max_ range, 1.0–24.0 h) and was similar on Day 1, Day 8, and Day 28 of Cycle 1. The results are consistent with the result of SDS sachet evaluated in the FIH study of BEZ235 in Western Patients [9]. Due to the long *T*_max_, the elimination half-life (*T*_1/2_) was not well characterized because of the absence of samples collected between 8 and 24 h post-dose (Table 6). Overall, the *C*_max_ and AUC appeared to increase in a dose-dependent manner.

**Table 6:** Summary of the primary PK parameters of BEZ235

Timepoint/dose level/statistics	AUC_last_ (ng h/mL)	AUC_inf_ (ng h/mL)	AUC_0 − 24_ (ng h/mL)	*C*_max_ (ng/mL)	*T*_max_ (h)
**Cycle 1 Day 1**					
400 mg qd					
*n*	3	2	3	3	3
Median	1036	978	1038	135	4.0
Min/Max	821/13,782	895/1061	821/13,869	69.9/1000	4.0/4.0
800 mg qd					
*n*	3	1	3	3	3
Median	2558	1488	2558	154	8.0
Min/Max	1433/6096	1488/1488	1438/6096	150/466	4.0/8.0
1000 mg qd					
*n*	6	3	4	6	6
Median	4492	1407	4334	472	5.0
Min/Max	869/19,292	890/7301	869/19,265	106/970	1.0/8.0
1200 mg qd					
*n*	5	1	3	5	5
Median	6420	2292	6417	403	6.0
Min/Max	2261/25,472	2292/2292	2264/15,775	203/1500	4.0/23.4
1400 mg qd					
*n*	5	1	2	5	5
Median	10,224	1286	13,736	733	8.0
Min/Max	1208/37,073	1286/1286	1215/26,258	87.7/1940	4.0/24.0
400 mg bid					
*n*	7	2	4	7	7
Median	2283	1995	1755	301	6.0
Min/Max	606/7719	1402/2587	606/5450	84.6/1150	1.0/8.0
**Cycle 1 Day 8**					
400 mg qd					
*n*	3	2	3	3	3
Median	7349	14,315	7351	754	4.0
Min/Max	4311/17,295	7657/20,974	4310/17,465	314/1470	4.0/8.0
800 mg qd					
*n*	3	1	3	3	3
Median	17,327	19,866	17,327	1170	6.0
Min/Max	8497/19,617	19,866/19,866	8497/19,626	609/1700	4.0/8.0
1000 mg qd					
*n*	7	3	3	7	7
Median	9347	8301	8261	912	6.0
Min/Max	482/46,393	496/9469	482/9347	76.1/2850	1.0/8.0
1200 mg qd					
*n*	4	1	4	4	4
Median	33,484	14,438	34,179	1810	4.0
Min/Max	12,760/46,522	14,438/14,438	12,840/46,522	1130/2410	2.0/4.0
1400 mg qd					
*n*	5	0	3	5	5
Median	35,882	–	36,375	2480	4.0
Min/Max	13,688/74,490	–	26,544/40,807	720/3910	3.9/7.9
400 mg bid					
*n*	6	1	5	6	6
Median	9702	2786	10,677	1046	4.0
Min/Max	2616/52,196	2786/2786	2616/53,881	455/6070	2.0/12.0
**Cycle 1 Day 28**					
400 mg qd					
*n*	3	3	3	3	3
Median	8916	10,192	8916	795	4.0
Min/Max	7756/25,942	7947/29,223	7769/26,182	600/1860	4.0/4.0
800 mg qd					
*n*	3	2	3	3	3
Median	24,870	13,540	24,955	1980	2.0
Min/Max	417/26,167	428/26,651	417/26,167	41.2/2190	1.0/4.0
1000 mg qd					
*n*	4	2	2	4	4
Median	4804	3769	3736	548	4.0
Min/Max	641/11,714	649/6889	641/6831	153/935	1.0/6.0
1200 mg qd					
*n*	2	0	1	2	2
Median	37,128	–	43,429	2170	5.9
Min/Max	31,005/43,252	–	43,429/43,429	1720/2620	3.9/8.0
1400 mg qd					
*n*	4	3	4	4	4
Median	18,270	30,688	18,343	1506	4.0
Min/Max	4394/36,862	7815/45,815	4394/36,862	317/2510	4.0/6.0
400 mg bid					
*n*	4	1	3	4	4
Median	34,083	2663	29,372	3650	4.0
Min/Max	2493/80,497	2663/2663	2493/40,839	518/8290	1.0/6.0

*AUC* area under the curve, *bid* twice daily, *C*_*max*_ maximum observed serum concentration after drug administration, *PK* pharmacokinetic, *T*_*max*_ time to reach C_max_, *qd* once daily

### Biomarkers

Sequencing results were obtained for nine tumor samples; no mutations of PIK3CA or PTEN were detected. One tumor sample was declared PTEN-negative, and in another patient, 40% of the tumor cells were PTEN-negative. Because of administrative problems, limited analysis was done from the collected small number of samples; no correlation with the clinical efficacy was observed and no further conclusions could be drawn.

Analysis of pS6 in normal skin was performed in 6 patients treated with 400 mg bid. Reduction was observed in 50% of the samples (3/6). Two of these three patients had SD until Cycle 4 or 5 with dose reductions up to 200 mg.

## Discussion

This study evaluated the safety and tolerability of BEZ235 doses from 400 mg to 1400 mg in Japanese patients with solid tumors. Each one out of six evaluable patients experienced DLTs of grade 2 allergic reaction at the 1200 mg dose level and grade 4 thrombocytopenia at the 1400 mg dose level. Thrombocytopenia was also reported as a DLT but allergic reaction was not reported as DLT in the FIH study in Western oncology patients. The patient diagnosed with allergic reaction had developed grade 1 rash and stomatitis. The study treatment was resumed after AEs were resolved, and on the same day, the patient experienced grade 1 fever. Fever was resolved with medication; however, on the next day, grade 3 rash was developed. These events of fever and rash that spread suddenly when study treatment was resumed, were judged as allergic reaction. Fever and rash were frequently reported AEs in different clinical studies of BEZ235, but there is no information about worsening of these events after re-exposure to study treatment. The single occurrence of this allergic reaction as DLT in our study does not allow conclusions about ethnic differences in the safety profile of BEZ235.

While the rates of DLTs were similar at both 1200 mg qd and 1400 mg qd, the safety of 1400 mg qd was considered less favorable (Table 2). Although the frequency of the DLT did not fulfil the criteria of the MTD prespecified in the protocol (the highest drug dose that causes DLT in no more than one out of six patients in Cycle 1), the maximum clinically tolerable dose of single-agent BEZ235 at a qd dosing schedule was determined to be 1200 mg qd, considering the observed toxicities and dose reductions in Cycle 2. The dose of 1000 mg qd was considered as the RD because it was the highest dose that did not cause any DLT.

In the FIH study of BEZ235 SDS sachet in Western oncology patients, only one DLT (grade 3 thrombocytopenia) was observed at 1600 mg; however, 50% of the patients (3/6) treated with BEZ235 at the dose of 1400 mg experienced DLTs including asthenia, fatigue, hyperglycemia, diarrhea, and mucositis (all grade 3). Eventually, 1200 mg was declared as the MTD based on the results from Bayesian Logistic Regression Model (BLRM), in which two DLTs (grade 3 vomiting and grade 2 nausea) were reported in six evaluated patients. Considering the safety profile observed in other clinical studies, 1000 mg was determined as an appropriate RD for further studies [10]. These findings support the results of our study [11, 12].

The bid dosing schedule had been introduced with an expectation to improve the absorption, and hence, the daily exposure of BEZ235. In addition, the gastrointestinal toxicities considered to be caused by local irritation of the gastrointestinal tract with qd dosing were expected to be mitigated through the division of the total daily dose. Considering the available strength at that time, only the 400 mg bid dose was investigated in this study, as it was the expected RD for the bid regimen based on the preliminary data from the clinical study in Western oncology patients. In that study, the preliminary results showed that two DLTs were observed at the 600 mg bid dose level, and 400 mg bid was considered as a tolerable dose in this study. Finally, the MTD and RD of BEZ235 in the clinical phase I study in Western oncology patients were declared as 300 mg bid each [12]. In our study, one out of six patients treated with 400 mg bid experienced a DLT of grade 2 liver dysfunction. Although the limited number of bid dose levels evaluated in this study does not allow further conclusions, this result of 400 mg bid is considered to meet the MTD definition as the highest dose level that causes DLT in no more than 1 of 6 patients. Overall, no major differences were observed between the safety profiles of the qd and bid groups. However, gastrointestinal toxicities of grade 3 or 4, such as nausea, tended to be more frequent in the 400 mg bid group compared with the 800 mg qd group, even though the total daily dose of 800 mg qd and 400 mg bid were the same, and the tolerability of 800 mg qd BEZ235 tended to be better than that of 400 mg bid. No patient in the 800 mg qd group reported dysphagia, but three out of eight patients complained of dysphagia in the 400 mg bid group. This could be because of the study medication being poorly soluble and difficult to swallow at the doses administered in this study. The results are similar to the phase I study in Western oncology patients, in which, bid dosing schedule did not result in decreased rates of toxicity compared to qd [12]. Consequently, other clinical studies of BEZ235 with bid dose also showed considerable toxicity [13].

Following oral administration at doses between 400 mg qd and 1400 mg qd, BEZ235 showed high inter- and intra-patient variability with different exposures. Once-daily dosing schedule of multiple formulations including hard gelatin capsule, SDS capsule, and SDS sachet were investigated in a clinical trial in Western oncology patients. However, across all formulations and dose levels tested (≥ 100 mg), BEZ235 displayed low bioavailability, with a moderate absorption rate, a less than proportional increase in exposure, and large inter-patient and intra-patient variability [9]. After bid administration, slow absorption and large inter-patient and intra-patient variability were observed. The variability in PK observed in this study in Japanese oncology patients was consistent with the results from the studies in Western oncology patients. The slow absorption and the low solubility of BEZ235 support a hypothesis of variable bioavailability. Another possibility is the impact of food intake. The FIH study of BEZ235 indicated that fed condition resulted in a higher exposure at 300 to 400 mg/day compared to fasting conditions at the same dose levels [9]. These results indicated that PK of BEZ235 is sensitive to the effect of food. In this study, patients were instructed to take BEZ235 after breakfast but without strict requirements regarding the food contents. Therefore, the difference in food condition between patients and study day might have affected the PK of BEZ235.

No patient achieved CR or PR at either dosing schedule. SD was observed as the best response in 14 out of 27 and two out of eight evaluable patients in the qd and the bid groups, respectively. Analysis of the pS6 status by a well-validated methodology in the 400 mg bid group revealed that 50% of the patients (3/6) showed a reduction in pS6 after treatment. Two out of three patients showed SD until Cycle 4 or 5 with dose reduction down to 200 mg. A tendency toward a more pronounced reduction in pS6 was observed in patients showing SD over four cycles (data not shown). Similarly, the mTOR inhibitor everolimus was shown to induce changes in pS6 in skin biopsies of patients with advanced solid tumors [14]. These results seemed to indicate a potential use of pS6 as a surrogate marker of pharmacodynamics of BEZ235. However, there is still no data to indicate the correlation of pS6 expression between tumor and skin, and no dose-related changes could be analyzed in this study. Further studies on pS6 in skin as a surrogate marker are necessary.

In conclusion, the MTD based on the standard 3 + 3 method was not reached in this study; however, 1200 mg was declared to be the maximum clinically tolerable dose for Japanese oncology patients using a qd dosing schedule. The dose of 1000 mg, which was the highest dose that did not cause any DLT, was determined as the RD. In addition, the tolerability of 400 mg BEZ235 with bid dosing was confirmed in Japanese oncology patients. The inter-individual variability of BEZ235 plasma concentrations in Japanese patients was high; however, plasma concentrations were consistent with the range reported in previous studies in Western oncology patients [9]. These results were comparable to the conclusions of the global phase I study of BEZ235.

Although BEZ235 as a single agent and in combination with other antineoplastic agents were investigated in various clinical studies, consideration of the more potent inhibition of mTOR than PI3K and highly variable PK profile of BEZ235 contributed to the discontinuation of further development in oncology indications [9].

## Electronic supplementary material

Below is the link to the electronic supplementary material.

Supplementary material 1 (DOCX 25 KB)
