# Supplementary material for: A phase I study of single-agent BEZ235 special delivery system sachet in Japanese patients with advanced solid tumors
Source: Cancer Chemother Pharmacol. 2018 Nov 16;83(2):289–99. doi: 10.1007/s00280-018-3725-2 (PMC6394493; doi:10.1007/s00280-018-3725-2)
Supplement: Supplementary file 1 — Supplementary material 1 (DOCX 25 KB) [file 280_2018_3725_MOESM1_ESM.docx]

**A phase I study of single-agent BEZ235 special delivery system sachet in Japanese patients with advanced solid tumors**

Masanori Toyoda,^1^ Koichiro Watanabe,^2†^ Taro Amagasaki,^3^ Kazuto Natsume,^3^ Hiromi Takeuchi,^3^ Cornelia Quadt,^4^ Kuniaki Shirao,^2^ Hironobu Minami^1^

^1^Division of Medical Oncology/Hematology, Department of Medicine, Kobe University Hospital, Kobe University Graduate School of Medicine, Kobe, Japan; ^2^Department of Medical Oncology & Hematology, Oita University Faculty of Medicine, Oita, Japan; ^3^Novartis Pharma K.K., Tokyo, Japan; ^4^Novartis Pharma AG, Basel, Switzerland

**^†^Present address:** Department of Medical Oncology, Kouseiren Tsurumi Hospital, Oita, Japan

**Journal name:** Cancer Chemotherapy and Pharmacology

**Correspondence to:**

Hironobu Minami, MD, PhD Med Sci

E-mail: [hminami@med.kobe-u.ac.jp](mailto:hminami@med.kobe-u.ac.jp)

**Table S1** Criteria for defining DLTs

| Toxicity | Any of the following criterion |
| --- | --- |
| Hematologic | ≥ CTCAE grade 3 neutropenia for > 7 consecutive days^.^ |
|  | CTCAE grade 3 thrombocytopenia for > 7 consecutive days^.^ |
|  | CTCAE grade 4 thrombocytopenia |
|  | Febrile neutropenia (ANC < 1.0×10^9^/L, fever ≥ 38.5°C) |
|  | ≥ CTCAE grade 3 anemia if judged to be a hemolytic process secondary to the study drug^a^ |
|  | ≥ CTCAE grade 3 lymphopenia if clinically significant^b^ |
| Renal | Serum creatinine ≥ 2.0×ULN to ≤ 3.0×ULN for > 7 consecutive days |
|  | ≥ CTCAE grade 3 serum creatinine |
| Hepatic^c^ | ≥ CTCAE grade 3 total bilirubin |
|  | CTCAE grade 3 AST or ALT for > 7 consecutive days^.^ |
|  | CTCAE grade 4 AST or ALT |
| Metabolic/Laboratory | ≥ CTCAE grade 3 asymptomatic amylase and/or lipase (asymptomatic) not reversible to ≤ CTCAE grade 2 within 7 days |
| Endocrine | Grade 2 hyperglycemia (confirmed with a repeat FPG within 24 hours) which cannot be resolved to grade 0 or baseline in ≤ 14 consecutive days |
|  | ≥ Grade 3 hyperglycemia (confirmed with a repeat FPG within 24 hours) |
| Cardiac | Cardiac toxicity ≥ CTCAE grade 3 or cardiac event that is symptomatic or requires medical intervention |
|  | Troponin ≥ CTCAE grade 3 or clinical signs of cardiac disease, such as unstable angina or myocardial infarction |
|  | > 20% decrease in heart rate from baseline to a value less than 60 bpm or symptomatic bradycardia |
| Pancreatitis | ≥ CTCAE grade 2 |
| Dermatologic | Any skin toxicity resulting in interruption^d^ of BEZ235 for > 7 consecutive days |
| Ophthalmologic (eye) | New onset retinal pigment epithelium change in the retina seen by clinical examination or by funduscopy or by fundus photography |
|  | A progressive reduction in visual acuity from baseline defined as loss of 10 letters or more as measured by Early Treatment Diabetic Retinopathy Study charts or 2 lines or more on any Snellen equivalent or Landolt chart |
|  | Other changes in ocular examination potentially attributable to BEZ235, including but not limited to, ocular inflammation, cataract changes, optic disc pallor, or corneal clouding |
| Phototoxicity | Any symptom experienced by the patient suggestive of a photosensitizing reaction |
| Other AEs | ≥ CTCAE grade 3 AEs (excluding ≥ CTCAE grade 3 elevations in alkaline phosphatase) |
|  | ≥ CTCAE grade 3 vomiting or ≥ CTCAE grade 3 nausea despite the use of standard anti-emetics |
|  | ≥ CTCAE grade 3 diarrhea despite the use of optimal antidiarrheal treatments |
| ^a^≥ CTCAE grade 3 anemia will not be considered as a DLT unless judged to be a hemolytic process secondary to the study drug ^b^≥ CTCAE grade 3 lymphopenia will not be considered as a DLT unless clinically significant ^c^For any grade 3 or 4 hepatic toxicity that does not resolve within 7 days to ≤ grade 1 (or ≤ grade 2 if liver infiltration with tumor present), an abdominal CT scan has to be performed to assess if it is related to disease progression  ^d^Not treating at the prescribed regimen is considered as an interruption  A single patient is assumed not to tolerate the dose if he/she experiences at least one DLT  Disease related symptoms will not define the DLT | |

*AE* adverse event, *ALT* alanine aminotransferase, *ANC* absolute neutrophil count, *AST* aspartate aminotransferase, *bpm* beats per minute, *CT* computed tomography, *CTCAE* Common Terminology Criteria for Adverse Events, *DLT* dose-limiting toxicity, *FPG* fasting plasma glucose, *ULN* upper limit of normal
